# Influence of Aging Temperature on Mechanical Properties and Structure of M300 Maraging Steel Produced by Selective Laser Melting

**DOI:** 10.3390/ma16030977

**Published:** 2023-01-20

**Authors:** Stepan Kolomy, Josef Sedlak, Jan Zouhar, Martin Slany, Marek Benc, David Dobrocky, Igor Barenyi, Jozef Majerik

**Affiliations:** 1 Faculty of Mechanical Engineering, Brno University of Technology, 616 00 Brno, Czech Republic; 2 Department of Mechanical Engineering, Faculty of Military Technology, University of Defence in Brno, 602 00 Brno, Czech Republic; 3 Department of Engineering, Faculty of Special Technology, Alexander Dubcek University of Trencin, Pri Parku 19, 911 06 Trenčín, Slovakia

**Keywords:** maraging steel, selective laser melting, aging, microhardness, microstructure

## Abstract

This paper deals with the study of high-strength M300 maraging steel produced using the selective laser melting method. Heat treatment consists of solution annealing and subsequent aging; the influence of the selected aging temperatures on the final mechanical properties—microhardness and compressive yield strength—and the structure of the maraging steel are described in detail. The microstructure of the samples is examined using optical and electron microscopy. The compressive test results show that the compressive yield strength increased after heat treatment up to a treatment temperature of 480 °C and then gradually decreased. The sample aged at 480 °C also exhibited the highest observed microhardness of 562 HV. The structure of this sample changed from the original melt pools to a relatively fine-grained structure with a high fraction of high-angle grain boundaries (72%).

## 1. Introduction

Additive manufacturing (AM) is a process for creating three-dimensional objects based on digital data. The difference between AM and machining, during which material is removed, is that in an AM process, material is gradually added. The final component is then created from thin layers laid one on top of another [1]. AM uses fine powders to create the required components, which is similar to powder metallurgy. The usage of initial powders instead of pre-cast alloys provides the researcher/producer with the ability to combine unique properties of individual elements and to create a wide range of (pseudo)alloys [2,3,4]. Using this technology, it is possible to produce complex shapes that cannot be produced by conventional casting, forming, or chip machining [5,6]. The use of additively manufactured components is almost unlimited. It is used in the automotive and aviation industries, as well as in healthcare and fashion [7,8,9].

In conventional forming processes, which also make it possible to manufacture a variety of components with numerous geometries but which work mostly with castings, plastic deformation is the main factor affecting the structures and properties of the materials (the imposed deformation can vary from relatively low values, such as during rolling [10], to very high, such as for severe plastic deformation processes [11,12,13]). AM makes it possible to produce different kinds of materials, the structures and mechanical properties of which can change depending on the printing parameters, strategies, heat treatment, chemical heat-treatment, etc., that have been used. The mechanical properties of the material and its service life can be effectively changed by appropriate heat and chemical-heat treatment [14,15].

Researchers (e.g., [16,17,18]) have analyzed nickel-based alloys manufactured by selective laser melting (SLM) with different hatching strategies. It was found that the resulting texture of the material can be influenced by changing the hatch strategy and the appropriate combination of power, hatch distance, and scanning speed. Others (e.g., [19,20,21]) investigated Inconel 718 prepared via AM. The results showed that bidirectional scanning without and with 90° rotation for each layer produces a bimodal columnar grain structure. The Ti-6Al-4V alloy produced by AM was also studied (e.g., [22,23,24]); different scanning strategies were documented to result in different energy distributions. The microstructure and crystallographic texture showed that a hexagonal scanning pattern sample yields a columnar primary β phase structure, while a chessboard pattern sample exhibits equiaxed morphology. Guan and Wang [25] investigated the effect of scanning strategies on surface morphology, defect reduction, and the mechanical properties of aluminum alloy samples produced by SLM. They found that layer-by-layer re-melting causes precipitation within the crystal lattice, leading to increased mechanical properties. Nong and Zhou [26] observed the effects of scanning strategies on the microstructure, surface texture, and mechanical properties of 15-5PH steel samples produced by the SLM method. The resulting structure showed fine grains and different proportions of austenite and martensite. Other researchers [27,28,29,30] have studied the effects of total energy density, scanning strategy, scanning speed, layer thickness, laser power, and hatch distance on the microstructure and mechanical properties of 316L stainless steel samples produced by SLM. It was found that laser power has a more significant effect on the mechanical properties than the scanning speed. Guo et al. [31] reported the effects of scanning strategy on grain size, element segregation, mechanical properties, and hot behavior of a high entropy alloy (FeCoCrNiMn) produced by SLM. The results showed that an alloy produced by the 45°-rotation-scanning strategy has a higher amount of hot cracking than the 67°-rotation-scanning strategy. Liu et al. [32] analyzed the effect of scanning strategy on the microstructure and mechanical properties of a ferritic martensitic steel produced by SLM. They concluded that a scanning strategy without subsequent rotation leads to higher yield strength and higher elongation than the strategy with 90° rotation. Deirmina, et al. [33] investigated the microstructure of 1.2344 tool steel produced by the SLM method. The as-printed microstructure consisted of a partially tempered column and a much higher amount of residual austenite compared to a quenched steel.

High-strength steels are known as maraging steels. The name of these steels is derived from “martensite aging,” i.e., hardening of martensite. These are highly alloyed steels containing very small amounts of carbon (max. 0.03 wt.%). The main elements forming maraging steels are Ni, Co, Mo, Ti, Al, and Cr. The structure, relative density, and mechanical properties are dependent on the printing direction [34], strategy [35], and process parameters [36]. Król et al. [37] investigated the influence of process parameters on the resulting material density, microstructure, and hardness. The results showed that the highest relative density was 99.30%. Mao et al. [38] addressed the same topic and achieved a relative density of 99.45%. Casalino et al. [39] obtained a relative density higher than 99%. Andronov et al. [40] dealt with the optimization of individual 3D-printing process parameters for M300 maraging steel to achieve a layer thickness of 100 µm. The authors showed that, when using this layer thickness, the resulting relative density is 99.9% and, at the same time, 3D printing is more productive. They produced a material with a final relative density of 99.98%. Laakso et al. [1] optimized 3D-printing parameters to acquire the lowest possible porosity. The final porosity was minimized to 0.09%. The goal was to obtain an isotropic material with the highest possible material density that would withstand high loads and cyclic fatigue [1]. For a better understanding of the effect of AM on the resulting mechanical properties, it is possible to perform an analysis of anisotropic properties [41].

The mechanical properties of maraging steels can be increased by applying (a combination of) heat treatments. The typical process consists of two individual treatments: during the initial heat treatment, Ni, Mo, and Ti alloying (additive) elements dissolve into the matrix, and during subsequent aging, a fine dispersion of hard Ni3Mo, Fe2Mo, and Ni3Ti precipitates is formed [37]. The size of the precipitates can be as fine as 50 to 150 nm [42]. High-strength mechanical properties are achieved due to this effect [43]. The effect of heat treatment on the microstructure and mechanical properties of 3D printed high-strength steel samples has been investigated elsewhere (e.g., [44,45,46,47,48,49,50]). It was found that the mechanical properties increased after combinations of solution treatment and subsequent aging treatment. Kim et al. [51] found that the use of solution annealing was not necessary to achieve the maximum hardness of 3D-printed, high-strength steel. Kannan et al. [42] achieved a good combination of strength and deformation properties after direct aging treatment at a temperature of 440 °C/6 h. It should be stressed, however, that heat treatment alone is usually not able to fully eliminate residual porosity. For this purpose, additional deformation treatment of materials produced by SLM is recommended; for example, the industrially usable rotary swaging method [52,53] can advantageously be used to eliminate porosity within steels produced via AM [54].

This paper deals with the study of the mechanical properties of M300 maraging steel subjected to different aging temperatures. The temperature range of the aging treatment was chosen in order to find conditions that could shorten the production cycle and reduce production costs, while maintaining favorably high mechanical properties of the material (especially microhardness and compressive yield strength). To the best of the authors’ knowledge, none of the previously published papers has examined the effects of the range of precipitation hardening temperatures between 420 °C and 520 °C at increments of 20 °C on M300 maraging steel samples produced by the SLM method, as defined by the process parameters determined further in experimental procedure. The temperatures were chosen due to the precipitation of small particles in this temperature range [49]. The focus of this study was on the structures acquired at these temperatures, as well as on detailed correlations of the aging temperatures with the microhardness achieved in the horizontal and vertical directions across the samples. The information collected on heat treatment, mechanical properties, and structures will be further used to design dynamically stressed parts with regard to machinability and production costs.

## 2. Experimental Procedure

**Figure 1:**
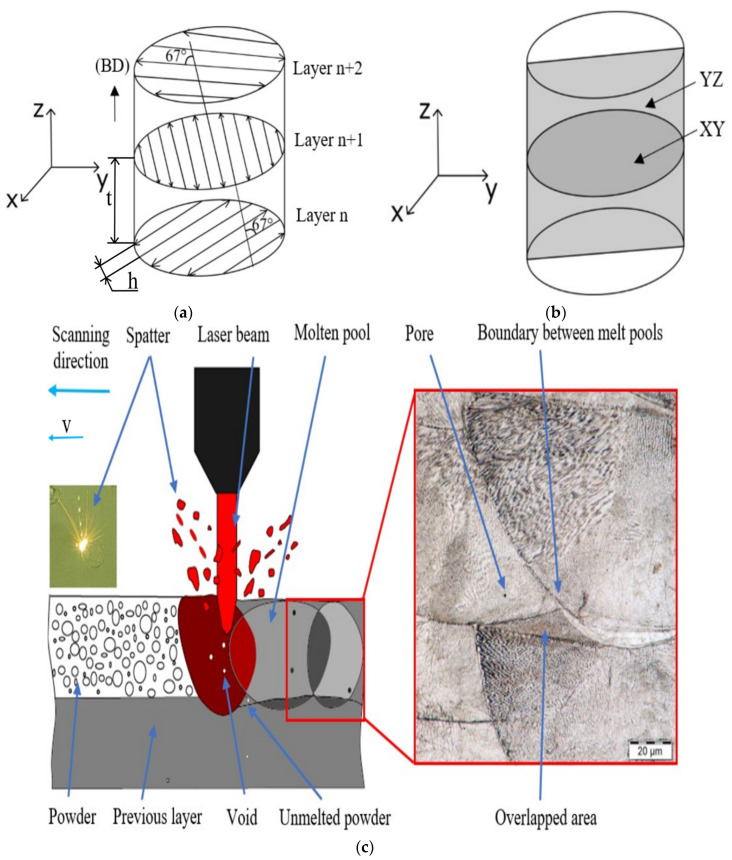
(**a**) Meander hatching pattern, 67° rotation after each layer, (**b**) Cross-section for microscopic analysis, and (**c**) grain-formation mechanism during the SLM process.

A metal powder of the maraging steel was used for production of the samples. The chemical composition of this steel is shown in Table 1. Using the RENISHAW RenAM 500E 3D printer (RENISHAW, New Mills, UK), cylindrical samples were built. The meander hatching pattern strategy with 67° rotation after each layer was used to manufacture the part (see Figure 1a). This strategy enables sufficient melting of the powder and formation of a structure with favorably low porosity [55]. The building direction (BD) is in the direction of the Z axis, the process parameters used are defined in Table 2. The value of the energy density used was calculated according to Equation (1) [35,56],

(1) $$E=\frac{P}{\mathrm{vht}}$$

where E is the laser energy density (J.mm^−3^), P is the laser power (W), v is the scanning speed (mm.s^−1^), h is the hatch spacing (mm), and t is the layer thickness (mm).

The printed samples usually feature residual stress after production. Nevertheless, this can be relaxed via heat treatment [57]. Heat treatment results in a reduction in residual stress, the precipitation of alloying elements, and also the creation of a uniform material structure [58]. For this reason, the printed samples were subjected to heat treatment, which took place under different conditions (see Table 3)

The process of heat treatment of the individual samples consisted of solution treatment (ST) at 840 °C/1h. ST was followed by aging treatment (AT) at temperatures of 420 °C/6 h to 520 °C/6 h. The heat treatment within this study was performed for 6 h, as it can be expected that with a reduced precipitation time (e.g., 3 h), there would be no significant changes in microhardness and compressive yield strength [42]. The samples were cooled in air to room temperature. After cooling, the microhardness of the individual samples was measured in the XY and YZ planes (see Figure 1b). These planes were chosen to describe the microhardness in the horizontal and vertical directions. Microhardness measurements were performed on a LECO 247 AT hardness tester for all the aging temperatures. The aim was to describe the dependence of microhardness on the aging temperature, because with appropriate heat treatment, production costs can be saved while the required mechanical properties can still be achieved. Figure 1c shows the mechanism of formation of the individual layers. The laser beam melts the powder locally and a molten pool is formed. The size of the melt pool depends on the energy density. The higher the energy density, the larger the melt bath and the better the melting of the metal powder [39]. Higher density can be achieved by increasing the laser power. However, higher laser power can cause more spatter, which can affect the surface layer [43]. The combination of individual process parameters affects the formation of the resulting structure and its porosity [36]. The resulting structure with less porosity will have higher resistance to wear and fatigue [59].

**Figure 2:**
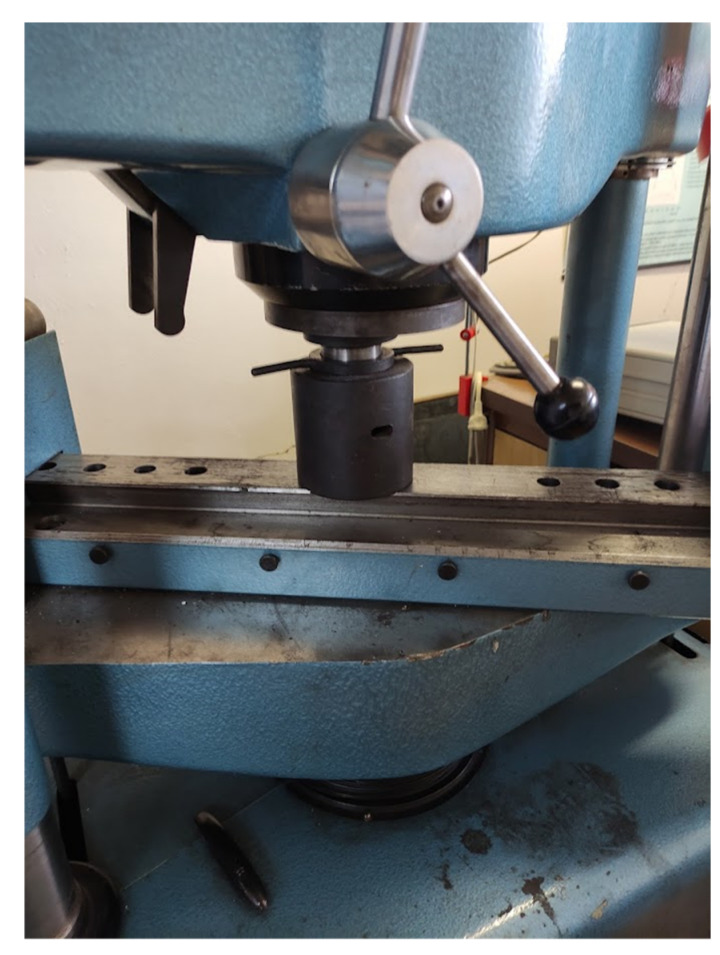
Hydraulic press used for compressive test.

The heat treatment process itself took place using the DIL 805A/D (Trencin, Slovakia) furnace. The printed sample had to be machined to the required dimensions. This was followed by mounting a G20P-A (Trencin, Slovakia) thermocouple to the surface of the sample, which scanned the temperature of the sample and controlled the heat-treatment process. The sample was then fixed between two spikes in the furnace space. After the heat treatment, the thermocouple was removed and the samples were placed in a special fixture (see Figure 2) in which the quasi-static test was performed. Compressive loading was carried out on a machine with an EDC 60 control unit (Brno, Czech Republic). The sample was pressed at a constant speed of 10 mm min^−1^. The TIRAtest v.2.1 program was used to obtain the data needed to determine the loading curves.

For the microscopic observations, the following preparation was carried out. A total of 14 samples were prepared. Seven of them were in the transverse (XY) and seven in the longitudinal (YZ) direction. Each sample was cut on a LECO MSX metallographic cut-off saw and pressed into a puck. The sample was subsequently ground and polished. Polishing was performed using 1 µm and 0.5 µm diamond pastes. Polishing was followed by etching with 2% nital solution. The prepared samples were at first observed using optical microscopy, i.e., using Olympus DSX500 (Olympus, Prag, Czech Republic) and KEYENCE VHX F microscopes (KEYENCE, Mechelen, Belgium). Furthermore, the chemical composition was determined using the Q4 TASMAN instrument (Bruker, Rudice, Czech Republic). For more detailed study of the microstructures, the following scanning electron microscopes were used: Tescan MIRA 4 (Tescan, Brno, Czech Republic) and Tescan Lyra 3 XMU FEG/SEMxFIB (Tescan, Brno, Czech Republic) with Symmetry EBSD detector. The scan step for the EBSD (electron-backscatter) scanning was 0.15 µm. The methodology that was used to evaluate the porosity is described in Grove and Jerram [60]; the ImageJ (version 1.53t) program was used for evaluations.

## 3. Results and Discussion

### 3.1. Mechanical Properties

**Figure 3:**
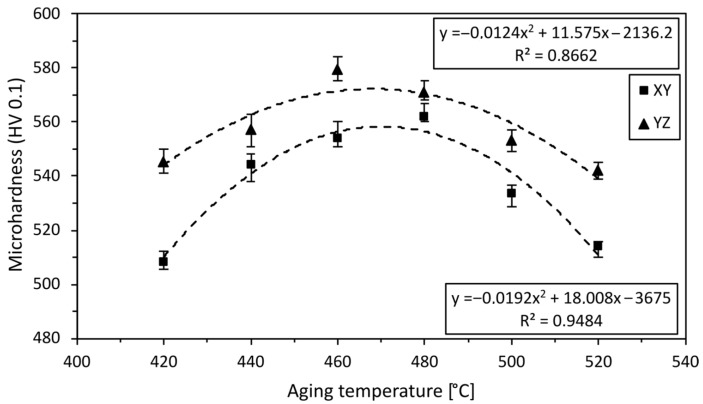
Dependence of Vickers microhardness on aging temperature (the microhardness is depicted in the XY and YZ planes).

Microhardness measurements in two perpendicular directions, i.e., in the XY (middle height of the sample) and YZ (along axis) planes (Figure 1b), were performed for each sample. These planes were chosen because the porosity is usually expected to be the highest in the vicinity of the axis, which is typically also related to the relatively lowest microhardness [54]. The microhardness was measured after heat treatment in an undeformed state. The resulting values are given as the average values calculated from 30 indentations. Figure 3. shows the correlation of the microhardness in the XY and YZ planes versus aging temperature. After 3D printing, the samples had a microhardness of 318 ± 10 HV, which is the lowest observed microhardness value. Solution annealing at a temperature of 840 °C for 1h resulted in an increase in microhardness to 356 ± 2 HV for all the samples. Figure 3 shows that the microhardness increased with increasing temperature, which is most probably due to the precipitation of fine precipitates [46]. It was found that the highest microhardness in the horizontal direction (562 HV) was achieved for the sample heat treated for 480 °C/6 h, and in the vertical direction this value was 579 HV for the 460 °C/6 h sample. This is an increase of 76.7% and 82%, respectively, compared to the as-printed state. At a temperature of 420 °C, the energy imparted by the heat treatment is not sufficient to induce precipitation to the same extent as at higher temperatures [42]. When the temperature is increased to 440 °C, 460 °C, and 480 °C, precipitation can develop to a greater extent due to higher imparted energy [61]. As the aging temperature increases, the precipitates tend to coarsen [49], which most probably also influenced the measured values presented here. Other factors that can influence the microhardness and cause differences between the values measured in the XY and YZ planes are grain size or the possible presence of martensite and residual austenite (further discussed in the following section). A higher martensite content generally causes higher microhardness, however, at the expense of ductility.

**Figure 4:**
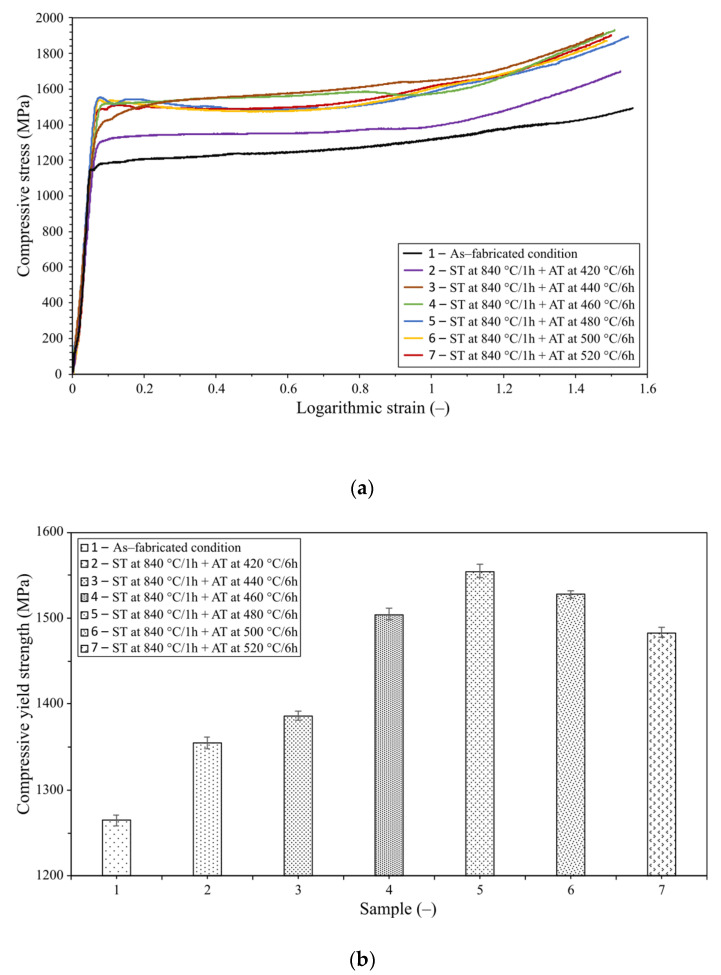
(**a**) Effect of different aging temperatures on compressive stress-strain curves, (**b**) compressive yield strength. For reference, measurements in the as-fabricated state are also included.

Figure 4a shows a graph depicting the dependence of compressive stress on logarithmic strain for all the examined samples (identical compression stroke was applied). The imposed strain was comparable for all the samples. The as-printed sample had the smallest compressive yield strength value of 1265 MPa. Conversely, the largest value of 1554 MPa was achieved for the sample that was aged at 480 °C/6 h (see Figure 4b depicting the values of compressive yield strengths for the individual samples). This means that the aging process resulted in a 22.8% increase in the compressive yield strength compared to the as-printed state. The results show that as the aging temperature increases, the hardness and compressive yield strength increase as well. This trend was observed up to a temperature of 480 °C, where the compressive yield stress decreases, most probably due to structural changes (see the following section). The higher the compressive yield strength, the higher the dynamical load that the components should be able to bear [43].

### 3.2. Microstructure Observations

**Figure 5:**
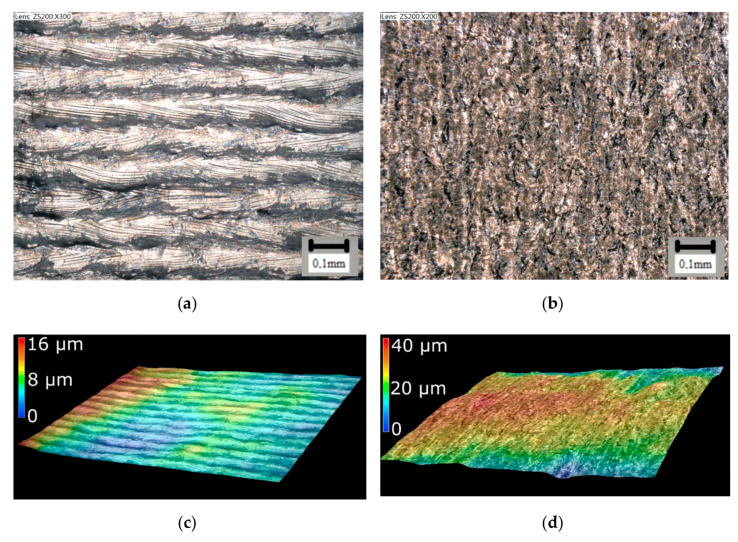
(**a**) Front view on SLM piece and (**b**) side view and (**c**,**d**) Rz surface roughness analysis.

3D printing is based on the principle of placing individual layers on top of each other until the entire part is fabricated. Figure 5a shows a view of the front surface of the sample directly after printing. In this figure, individual tracks that correspond to the hatch distance can be seen. Figure 5b shows a view of the cylindrical surface of the sample, and Figure 5c,d then show sample surface images created using a KEYENCE digital microscope. Obviously, the surface structure does not have the quality required to be considered a functional surface (Ra 10.1 µm, Rz 23 µm), and, therefore, it is necessary to machine these surfaces [62,63].

**Figure 6:**
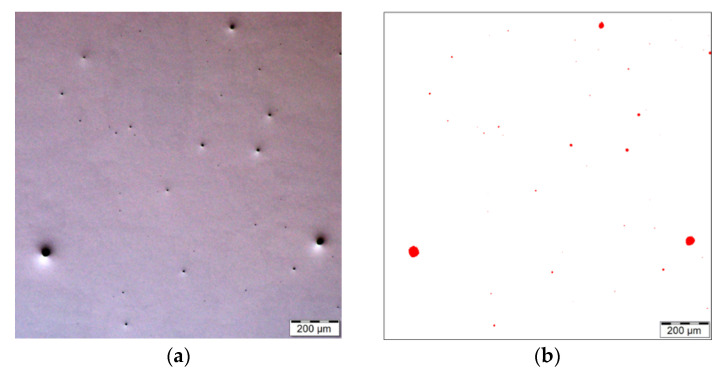

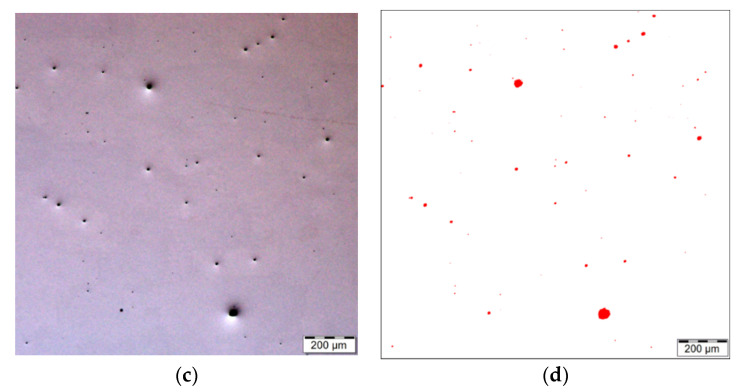
Light optical microscopy (LOM) images show porosity in the XY plane (**a**,**b**) and in the YZ plane (**c**,**d**) for the analyzed material; (**a**–**d**) correspond to sample 1 presented in Table 3.

The process parameters of 3D printing, i.e., laser power 400 W, layer thickness 0.04 mm, hatch spacing 0.095 mm, and scanning speed 830 mm.s^−1^, resulted in the creation of a structure with a residual porosity of 0.220% in the XY plane and 0.274% in the YZ plane (see Figure 6). The average pore size was 83.6 µm in the XY plane and 66.3 µm in the YZ plane. The porosity was investigated on polished sample 1 (as-fabricated). The presence of pores is most probably due to the presence of gas that remained trapped in the material during sintering of the individual layers [64,65]. Similar results were achieved, e.g., by Casalino et al. [39]; decreasing the porosity can result in increasing the mechanical and fatigue properties, because individual pores are possible stress concentrators.

**Figure 7:**
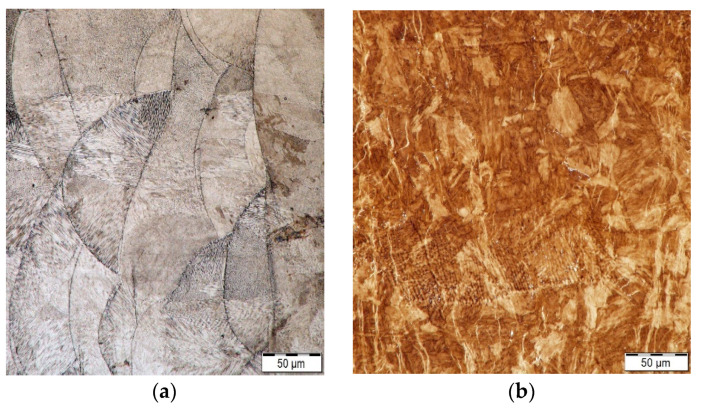
Light optical microscopy (LOM) images of (**a**) sample 1, (**b**) sample 5.

Detailed optical and electron microscopy analyses of two samples (Table 3)—sample 1 and sample 5 exhibiting the highest compressive yield strength—were further carried out. Figure 7a shows a LOM image taken from the as-fabricated sample 1. The structure evidently consisted of characteristic melt pools featuring individual grains (visible in numerous locations). On the other hand, the structure of sample 5 featured grains with characteristic martensitic laths (Figure 7b). This structural feature most probably contributed to the increase in the values of the mechanical properties achieved for this sample (as stated above).

**Figure 8:**
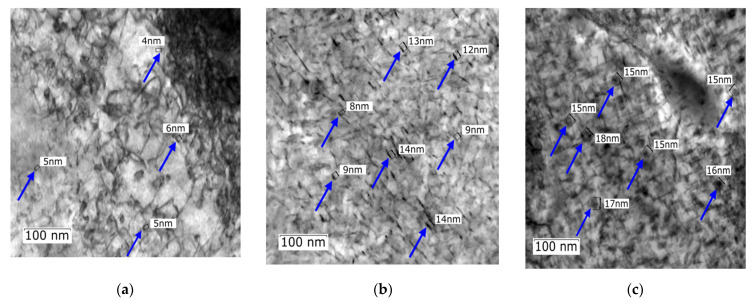
(**a**) Detailed TEM view of precipitate particles across the cross-section of sample 2 aged at 420°C/6h, (**b**) TEM view of sample 5 aged at 480 °C/6 h, (**c**) TEM view of sample 7 aged at 520 °C/6 h.

The areas of interest for TEM observations within sample 2, 5, and 7 included locations containing small precipitates. Detailed images of numerous precipitates formed across the examined XY plane within these samples can be seen in Figure 8a,c. The applied aging temperatures generally led to the formation of a large quantity of uniformly dispersed nanosized precipitates with needle-like and spheroidal shapes (indicated with dark blue arrows). These types of precipitates (especially the needle-like precipitates) were also observed by the authors of study [66]. The presence of precipitates, which are clearly visible within the structure, most probably contributed to the increased mechanical properties as well. Figure 8a shows a small number of needle-like shaped precipitates, which were about 4–6 nm in length. The density of nanoprecipitates was lower compared to samples 5 and 7 depicted in Figure 8b,c. The increased aging temperature not only led to the formation of a higher number of nanoparticles, but also to their larger size. Sample 5 (see Figure 8b) featured a higher number of precipitates corresponding to the higher aging temperature. The length of nanoprecipitates in sample 5 was higher (about 8–14 nm) than in sample 2 (sample 5 had the highest microhardness value in the XY plane after heat treatment). Clearly, the size of the nanoprecipitates gradually increased with increasing aging temperature from 420 °C to 520 °C. With increasing aging temperature, the precipitates tended to change their shapes from needle-like to spheroidal, which was also accompanied by an increase in size, i.e., 15–18 nm (see Figure 8c). The size and shapes of precipitates within sample 7 probably contributed to the decreased mechanical properties.

**Figure 9:**
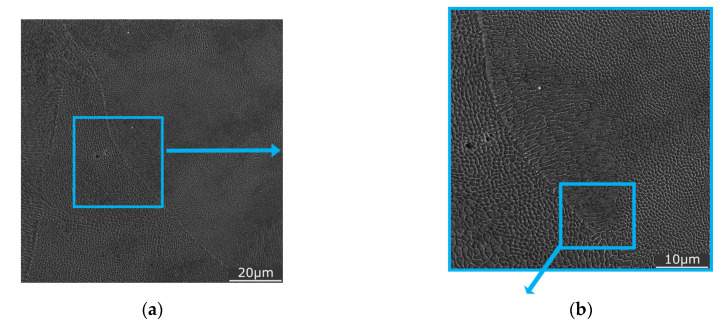

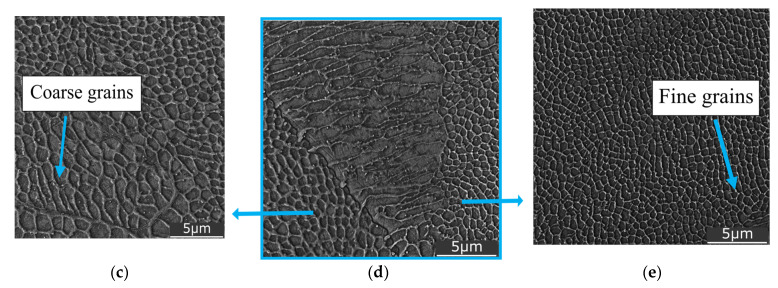
Detailed view of the microstructure of sample 1 (XY plane), (**a**,**b**,**d**) area of interest, (**c**) detail of coarse grains, (**e**) detail of fine grains.

For comparison, sample 1 was also subjected to detailed SEM observations. Figure 9 shows a detailed view of the structure of this sample (XY plane) featuring individual grains within the characteristic melt pools (as seen in Figure 7a). The sizes and shapes of the grains and the entire melt pools depend on the relationship between their orientations and the printing process. In other words, very high cooling rates and differences in thermal conductivity in different directions are the most important factors that influence the formation of the structure, which subsequently affects the mechanical properties [47].

**Figure 10:**
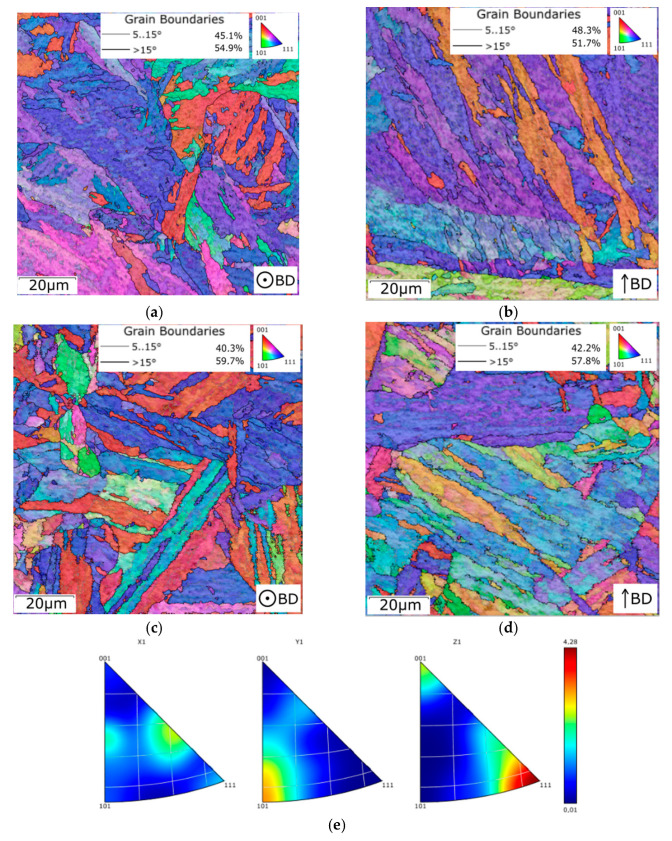

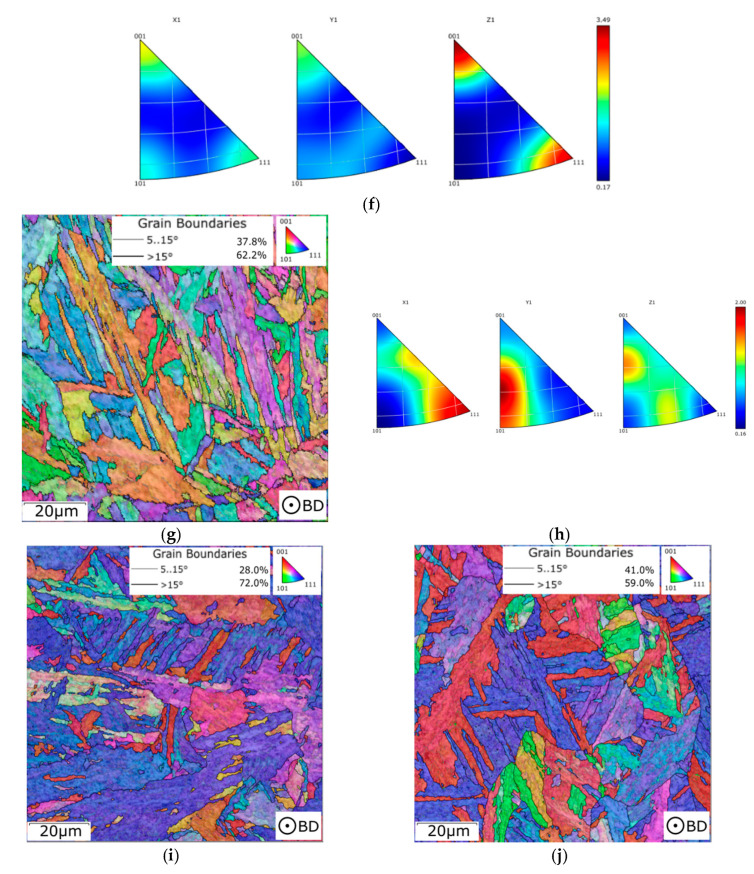

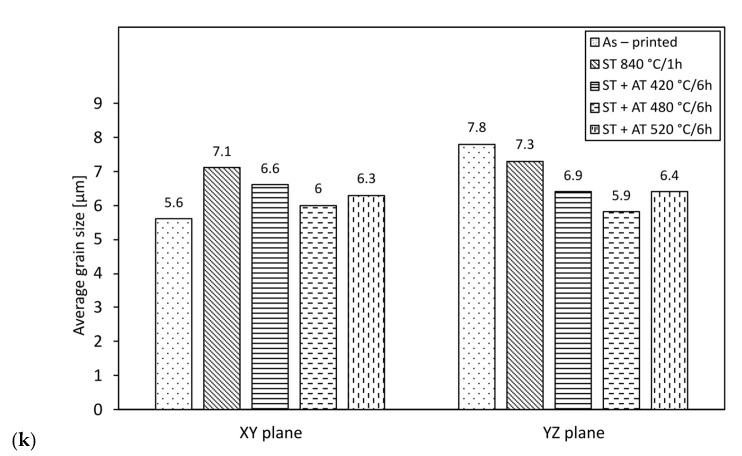
OIMs for sample 1 (**a**) XY plane and (**b**) YZ plane; sample after solution treatment (**c**) XY plane and (**d**) YZ plane. IPFs for sample 1, XY plane (**e**); sample after solution treatment, XY plane (**f**). OIM for sample 2, XY plane (**g**); IPFs for sample 2, XY plane (**h**). OIM for sample 5, XY plane (**i**); OIM for sample 7, XY plane (**j**); summary of average grain sizes in the XY and YZ planes (**k**).

Figure 10 shows orientation image maps (OIMs) acquired from transverse (XY plane) and longitudinal (YZ plane) sections of samples 1, 2, 5, and 7 (Table 3), as well as a sample after solution treatment. In the maps, the grains are depicted in colors corresponding to their individual orientations according to Miller indices (see the legend triangle in each figure). The grain orientations were evaluated parallel to the axis of the sample. In other words, the transversal scans (XY plane) are evaluated parallel to the z axis (|| z), while the longitudinal scans (YZ plane) are evaluated parallel to the x axis (|| x).

The as-fabricated sample 1 exhibited a typical structure as created by the 3D-printing process, i.e., corresponding to individual laser paths (hatch spacing). The average grain size for this structure differed slightly in the examined XY and YZ planes (examination based on OIM scans, limit for a high-angle grain boundary (HAGB) considered to be 15°). The avg. max. grain feret diameter in the XY plane of sample 1 was 5.6 µm, while for the YZ plane this value was 7.8 µm (the grain sizes for the examined samples are summarized in Figure 10k). For the evaluations, the maximum grain feret diameter was defined as the maximum distance between two points located within a single grain [67]. The grains within this sample also exhibited a strong tendency to form the <111>||z preferential orientation (Figure 10a,b). The heat treatment at 840 °C led to structure homogenization as regards the grain size; the corresponding values for the XY and YZ planes were 7.1 µm and 7.3 µm, respectively (based on Figure 10c,d). The treatment also yielded a decrease in the maximum texture intensity (compare the fiber texture intensities in Figure 10e,f depicting characteristic inverse pole figures (IPFs) for samples right after fabrication and after solution treatment). Because the differences between the structures in the XY and YZ planes diminished after the solution treatment, the results of structure analyses for aged samples 2, 5, and 7 are further graphically depicted only for the transversal XY plane.

Figure 10g depicting the XY plane OIM of aged sample 2, together with the IPF for the sample depicted in Figure 10h, shows that the texture intensity further decreased after the 420 °C/6 h aging treatment. However, the HAGB fraction increased to 62.2% (compared to 54.9% and 59.7% achieved for the as-fabricated and solution-treated samples, respectively). The grain size decreased as well to a value of 6.6 µm in the XY plane and 6.9 µm in the YZ plane. The difference in these values for both the measured planes was comparable to that observed for the solution-treated sample. After aging at 480 °C (Figure 10i), the HAGB fraction increased to the maximum observed level of 72%, and the avg. grain size slightly decreased to 6.0 µm in the XY plane and 5.9 µm in the YZ plane. These values were the most homogeneous of all the examined samples. The OIM also indicates that the increased temperature caused the texture to begin forming again. This trend was also visible within sample 7, aged at 520 °C (Figure 10j). This sample also exhibited a decrease in the HAGB fraction to 59% (comparable to the sample after solution treatment and to aged sample 2) and an increase in the avg. grain size to 6.3 µm in the XY plane and 7.8 µm in the YZ plane. The difference between these two grain-size values was the largest of all the examined samples and points to some (undesirable) grain growth due to the effect of the increased aging temperature.

Based on the acquired data, the changes in the mechanical properties were most probably influenced by the following principle factors: precipitation, grain size, and HAGB fraction. It was found that sample 5, aged at 480 °C, which exhibited the highest microhardness and also the highest compressive yield strength, featured a substantial amount of homogeneously distributed fine precipitates (Figure 8), as well as the highest observed volume fraction of HAGB. It also featured the lowest grain size with the most homogeneous distribution throughout the examined perpendicular planes. This sample also featured martensite within its structure (Figure 7b). The surface of such material (i.e., featuring the achieved microhardness) will most probably show favorable abrasion resistance and wear resistance (conversely, a material with lower hardness will not have such favorable wear resistance, but will most probably show higher ductility). Good surface quality and low porosity are also expected to increase resistance to dynamic cyclic loading. Selecting an optimized aging temperature is important from the viewpoint of the combination of favorable mechanical properties and production costs; temperatures above 500 °C are not suitable due to the higher expected production costs. Furthermore, such temperatures led to undesirable structural changes, such as grain growth, as proven by the presented analyses, resulting in decreasing mechanical properties. The texture and grain orientations most probably did not have a significant effect on the mechanical properties; however, these parameters are expected to play a significant role during plastic deformation—the effects of post-process plastic deformation on as-printed and aged samples are planned to be the main focus of our following study.

## 4. Conclusions

In this paper, the effects of selected aging temperatures on the microhardness, compressive yield strength, and structure of M300 maraging steel produced by the SLM method were studied. For the as-fabricated sample, the selected conditions of the SLM process resulted in a structure that showed 0.220% porosity along the transversal axis and 0.274% porosity along the longitudinal axis. The average grain size in the corresponding planes was 5.6 µm and 7.8 µm, respectively. After heat treatment, the grain size generally decreased, as did the texture intensity (these trends were observed up to an aging temperature of 480 °C, above which the trends reversed). Aging also promoted the development of precipitates and a martensitic phase within the samples.

The maximum microhardness across the sample and the maximum compressive yield strength were achieved for the sample subjected to 480 °C/6 h aging. This sample exhibited a dispersion of fine precipitates, the finest observed grains, a high volume fraction of high-angle grain boundaries, and the presence of martensite. A clear effect of texture on the mechanical properties of the aged samples was not observed. However, texture will most probably be an important factor influencing post-process deformation processing.

## Figures and Tables

**Table 1:** Chemical composition of fabricated samples using SLM.

Element	Ni	Co	Mo	Ti	Si	Mn	C	P	Fe
Q4 TASMAN Wt. %	18.42	8.86	4.65	0.70	0.01	0.04	0.02	0.02	66.40

**Table 2:** Process parameters used for fabrication of the parts using SLM.

Energy Density [J.mm^−3^]	Laser Power [W]	Scanning Speed [mm.s^−1^]	Laser Thickness [mm]	Hatch Spacing [mm]
127	400	830	0.04	0.095

**Table 3:** Experimental design of temperature treatment.

Number	1	2	3	4	5	6	7
Solution treatment	As-fabricated	840 °C/1 h	840 °C/1 h	840 °C/1 h	840 °C/1 h	840 °C/1 h	840 °C/1 h
Aging treatment	As-fabricated	420 °C/6 h	440 °C/6 h	460 °C/6 h	480 °C/6 h	500 °C/6 h	520 °C/6 h

## Data Availability

Not applicable.
